# An overhang-based DNA block shuffling method for creating a customized random library

**DOI:** 10.1038/srep09740

**Published:** 2015-05-26

**Authors:** Kosuke Fujishima, Chris Venter, Kendrick Wang, Raphael Ferreira, Lynn J. Rothschild

**Affiliations:** 1 University Affiliated Research Center, NASA Ames Research Center, Moffett Field, CA, USA; 2 NASA Education Associates Program (EAP), NASA Ames Research Center, Moffett Field, CA, USA; 3 Department of Bioengineering, Stanford University, Stanford, CA, USA; 4 Université Paris VII – Diderot, France; 5 NASA Ames Research Center, Moffett Field, CA, USA

## Abstract

We present an overhang-based DNA block shuffling method to create a customized random DNA library with flexible sequence design and length. Our method enables the efficient and seamless assembly of short DNA blocks with dinucleotide overhangs through a simple ligation process. Next generation sequencing analysis of the assembled DNA library revealed that ligation was accurate, directional and unbiased. This straightforward DNA assembly method should fulfill the versatile needs of both *in vivo* and *in vitro* functional screening of random peptides and RNA created with a desired amino acid and nucleotide composition, as well as making highly repetitive gene constructs that are difficult to synthesize *de novo*.

Synthetic biologists have developed various DNA synthesis and assembly methods to generate large DNA constructs that can reach the size of a whole bacterial genome with high precision^1^. Creating an accurate and flexible combinatorial DNA library is another key milestone for synthetic biology, given the need for screening functional DNA/RNA elements and protein aptamers using various *in vitro* selection methods^2^,^3^,^4^, as well as *in vivo* studies on genomic sequence variants^5^ and DNA barcoding of individual cells^6^. As the complexity of a library increases, it remains expensive to fully synthesize each DNA sequence.

Several methods have been developed for creating combinatorial DNA libraries with high complexity, but each method has drawbacks. Most of these are due to the biased representation of codons as well as the formation of unexpected restriction sites. Traditional site saturation approaches using random poly-NNK (K: G or T) libraries avoids two out of the three stop codons while covering all twenty amino acid codons, but the length of the randomized region is short and a fixed length^7^. The ordered assembly of multiple DNA building blocks gives flexibility in length but requires a successive restriction enzyme digestion and ligation^8^. Standard restriction enzyme leaves a scar sequence between each sequence, resulting in one or more fixed amino acids at every ligation site. Homologous recombination with PCR-based extension is another common assembly method but requires the preparation of long overlapping dsDNA or oligonucleotides that limits the flexibility of sequence design. Furthermore each additional PCR step increases mutation error, workload and cost of library preparation^9^. Recent advances in ink-jet–based microarray printing can offer millions of DNA sequences up to 200 bp in length at a reasonable cost^10^, but this method requires specialized instrumentation, and the quantity and length of the constructs are insufficient for the functional assays that deal with sequences on the order of 10^10^ to 10^13^.

In order to simplify the procedure and minimize the errors, we have developed an overhang-based DNA block shuffling method for combinatorial DNA library construction. The flexibility in both sequence design and length allows versatile downstream *in vitro* and *in vivo* analyses (Fig. 1a). We first designed 16 bp DNA blocks with various nucleotide overhangs and tested their efficiency during ligation. Palindromic dinucleotide pairs (i.e., GC-CG, AT-TA) were excluded to maintain directional ligation. Based on a visual examination of the electrophoretic analysis, all five dinucleotide overhang pairs displayed ladder-like ligated concatemers of up to 16 blocks and longer (Fig. 1b). As anticipated, a single nucleotide A-T overhang failed to generate long ligation products, while a G-C pair resulted in concatemers of up to 13 blocks, similar to the efficiency of dinucleotide overhangs. We also tested various oligo lengths ranging from 12 bp to 30 bp with overhang sequence fixed to an AG-TC dinucleotide pair. Ligation products were visually confirmed up to 1 kbp in length (Fig. 1c). In order to evaluate the performance of our assembly method, a total of 168 degenerate oligonucleotide pairs corresponding to 8288 DNA blocks were prepared. These oligonucleotides encoded hexapeptides consisting of only ten amino acids: Ala, Asp, Glu, Gly, Ile, Leu, Pro, Ser, Thr and Val. Degenerate oligonucleotides were designed by following the binary pattern rule^11^ to code for different hexapeptide secondary structures and were annealed to create dsDNA with AG-TC overhangs (Supplementary Table 2). In order to create a combinatorial gene library, DNA blocks were phosphorylated, mixed with 5′ and 3′ dsDNA adapter sequences containing restriction sites, and ligated in one-pot reaction (Supplementary Fig. 1a). The assembled gene library was further PCR amplified to extract and concentrate gene constructs that harbor 5′ and 3′ PCR adapters for downstream cloning. PCR products were further processed using TruSeq DNA kit and was sequenced by Illumina Miseq with 150 bp paired-end output (Illumina, San Diego, CA, USA). Out of 1,396,867 raw sequence reads, 64,019 sequences containing more than one unknown (N) base were discarded and low-quality ends of the reads were trimmed. Remaining 1,332,848 reads had an average Phred quality score peak around Q27 (99.8% accuracy), while quality across all bases have shown deterioration toward the end of the read (Supplementary Fig. 2 and 3). We further extracted 449,748 DNA block sequences containing adapter sequences on both ends to validate the quality of concatemers that will be used in the downstream *in vitro* and *in vivo* analyses. As a result, the majority of ligated DNA blocks had the expected lengths in multiple of 18 bp with a peak around 72 to 90 bp (4–5 blocks) and continuing up to 198 bp (11 blocks) in length, reaching the upper limit of the Miseq sequencing (Fig. 2a). DNA blocks with incorrect lengths accounted for 14.8% of the total reads, due to the original oligonucleotide quality, mis-annealing/ligation and PCR error. Among the sequences with the correct length, nonsense and missense mutations account for 2.2% and 7.7% of the reads respectively, resulting in stop codon insertion and incorporation of unintended amino acids (Fig. 2b). A total of 75.3% of the reads were correctly assembled to provide genes encoding polypeptides consisting of limited set of amino acids. Sequence accuracy declined with an increase in the numbers of DNA block concatemers due to the accumulation of errors but maintained an accuracy of more than 74% up to 8 blocks in length (Fig. 2c). We also counted non-redundant sequences within each concatemer to extrapolate theoretical number of genes generated. Assuming an initial input of 10^14^ DNA blocks (~320 pmol) for ligation, the number of non-redundant sequences for concatemers longer than 4 blocks can reach between 10^8^ to 10^13^ depending on their length (Supplementary Table 1), suggesting that this method can provide sufficient sequence variety for both *in vivo* and *in vitro* library screening.

Lastly, we checked the ligation bias that may have occurred during the overhang-based DNA block shuffling. We extracted a total of 1,111,779 DNA block combinations from the assembled sequences that had more than one block ligated. However, the sequence corresponding to helix 34 was completely missing (data not shown); thus, we concluded this was a result of unintended misplacement during the ligation step. A heat map of 167 by 167 DNA block combination classified by their coding protein secondary structure showed clear overrepresentation of DNA block corresponding to helices, but not limited to helix-helix ligation. Moreover, approximately 98% (163/167) of all DNA blocks were represented within one order of magnitude between 2000–20,000 times (Fig. 2e). These results suggest an unbiased feature of ligation reaction.

By constructing a customized random DNA library that encode proteins consists of only ten amino acids, we achieve to demonstrate accurate, directional and unbiased ligation properties of our novel overhang-based DNA block shuffling method. Critically, our approach maintains tight control over the amino acid representation in the library compared to the traditional poly-NNK method (Fig. 3). Thus, this method provides great flexibly for users to design original “DNA blocks” based on different applications (i.e., cell barcoding, ribozyme and peptide aptamer screening, constructing *de novo* proteins consist of limiting amino acid variation, etc.). As a proof of concept, we used low-priced standard oligonucleotides ($0.18/nt), which can be obtained through any commercial oligo synthesis company. The total cost for the 168 degenerate DNA blocks was approximately $1000, which is cost efficient given the flexibility of mix-and-matching DNA blocks to create a variety of combinatorial libraries. This method will benefit from the progress in the next generation oligonucleotide synthesis to further lower the initial cost of oligonucleotides. The simplicity of this method is advantageous for maintaining a high quality DNA library, since the error-producing steps are limited to annealing, ligation and PCR, resulting in 75.3% overall accuracy (Fig. 2b). Currently there are methods to reduce sequence error at the DNA level, through commercial enzymatic cocktails such as ErrASE (Novici Biotech, Vacaville CA, USA), and at the protein level, by performing pre-screening using a protein tag-coded spacer sequence to remove all the frame-shifted products^8^. These approaches can easily be combined with our method to further increase accuracy, especially for the longer concatemer products that are prone to error (Fig. 2c). One of the key advantages of our method is the efficient production of a combinatorial protein library with limited amino acid alphabets, which can be used to analyze early protein evolution^12^. Our library is also suitable in making a gene library of highly repetitive sequences with various lengths. Immediate applications could be the study of various repeat expansion disorders^13^, as well as the production of industrially and biomedically important proteins with repetitive segments^14^ such as spidroin (spider silk protein) and elastin-like proteins.

## Methods

### Oligonucleotides

All oligonucleotides were obtained from Elim Biopharmaceuticals, Inc. (Hayward, CA, USA) with standard purification at 200 μM concentration in salt-free di-water. A total of 168 degenerate oligonucleotide pairs, two adapter sequences and PCR primers used in this work are listed in Supplementary Table 2.

### Overhang-based DNA block shuffling

**Figure 1:**
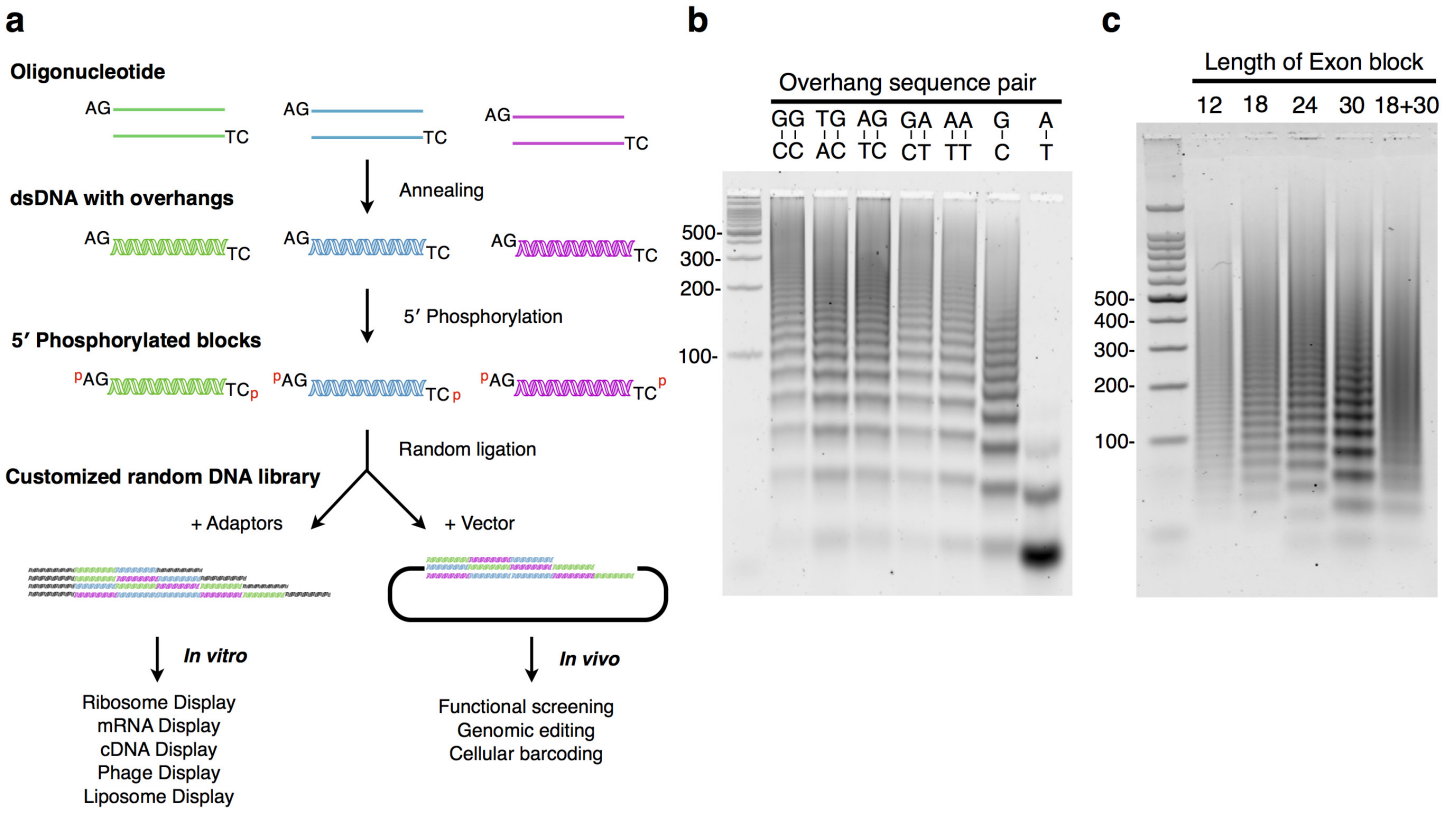
Overhang-based DNA block assembly method and design. (a) Each pair of complementary oligonucleotides was annealed to form a dsDNA with 5′ overhangs (in this case AG-TC), followed by 5′ phosphorylation and ligation with adapter DNA or a linearized plasmid to generate a combinatorial random DNA library. (b) DNA gel electrophoresis of DNA blocks with single or di-nucleotide overhangs ligated via our assembly method. (c) Assembly of DNA blocks with AG-TC overhang harboring various lengths (12 bp to 30 bp, including a mix of 18 bp and 30 bp blocks).

Each degenerate oligonucleotide pair was mixed in equal amount in a tube buffered with 10× H buffer (Promega, Madison, WI) to a final concentration of 80 μM oligo in 0.4× H buffer (serving as an annealing buffer). Oligonucleotides were denatured by heating at 94°C for 2 min on a Echotherm heat block (Torrey Pines Scientific Inc., Carlsbad, CA, USA) followed by gradual cooling and annealing at room temperature by moving the heat block from the heater to the bench top. Once the oligo mix reached room temperature, the tube was placed on ice to finalize the annealing step to generate DNA block with overhangs. Alternatively, a thermal cycler can be used to obtain programmed denaturation and stepwise cooling with a constant decrease of 10°C every 5 min. Annealed products (dsDNA with overhangs) were phosphorylated with T4 Polynucleotide Kinase (New England Biolabs, Ipswich, MA) in a 10× ligase buffer at 37°C for 1 h. Then, T4 DNA ligase (New England Biolabs) was directly added to the phosphorylation reaction mixture and incubated at room temperature for 2 h to yield the randomly ligated DNA block concatemers (Fig. 1a).

### Adapter sequence preparation and ligation

Two adapter dsDNA sequences (5′ and 3′) were prepared in a same way as DNA blocks, but only one side of the adapter dsDNA sequence contained phosphorylated overhangs in order to avoid circularization and self-ligation (Supplementary Fig. 1a). Adapter sequence can include, promoter, ribosome binding site, epitope tag, restriction site, RNA regulatory elements, etc., depending on the types of research. Alternatively, restriction digestion of *de novo* dsDNA or a plasmid using type II restriction enzymes (i.e., BtsCI, BseRI, BceAI), will generate long 5′ and 3′ adapter sequences with dinucleotide overhangs (Supplementary Fig. 1b and 1c). Adapter sequences with phosphorylated dinucleotide overhangs were mixed with phosphorylated DNA blocks in 1× T4 ligase buffer condition with a ratio of 1:8:1 (5′ adapter: DNA block: 3′ adapter) and incubated with T4 DNA ligase at room temperature for 2 h. Ligation can be also performed at 16°C overnight to increase the efficiency.

### DNA Sequencing

The ligation product was purified using MinElute Reaction Cleanup Kit (Qiagen, Limburg, Netherlands), and a small portion (10 ng) was PCR amplified with Platinum PCR Su perMix (Life Technologies, Carlsbad, CA, USA) with initial denaturation at 94°C for 30 seconds for melting, annealing at 52°C for 30 seconds and elongation at 72°C for 1 min, repeating 30 cycles (PCR cycles can be reduced down to 10 cycles depending on the initial DNA input and downstream experiment). PCR products were cleaned up using Wizard SV Gel and PCR Clean-Up System (Promega). TruSeq DNA kit (Illumina, San Diego, CA, USA) was applied to a total of 200 ng of PCR product to add Illumina-compatible adapters, including end-repair, A-tailing and ligation, to the library. 10 cycles of adapter-complement PCR was performed to enrich the sequence-able fragments in the libraries. Size selection was performed with standard ampure magnetic beads at 0.8× to remove small fragments including adapter dimers. The prepared DNA sample was processed with 150 bp paired-end sequencing using Miseq v3 chemistry.

### Sequence analysis

FASTQ output file was parsed using the online Galaxy platform^15^,^16^ (https://usegalaxy.org) for trimming, checking quality and converting file to a multi-FASTA format. First, FASTQ Quality Trimmer was used to remove low quality ends until a sliding window of 50 bp exceeded QC15. FastQC package was then used to obtain average Phred quality score per base (Supplementary Fig. 2) and per read (Supplementary Fig. 3). To extract high quality sequences with PCR adapter on both 5′ and 3′ ends, we further applied a strict rule that a read must contain at least 8 bp complete matches of the adapter sequences flanking the ligated concatemers. Extraction and count of high quality reads were performed on multi-FASTA file using our original Perl script program (Supplementary file 1). A heat map was constructed using a Heat Map Viewer module provided through the genomic analysis platform GenePattern^17^ (http://www.broadinstitute.org/cancer/software/genepattern/).

## Supplementary Material

Supplementary Information Supplementary materials

## Figures and Tables

**Figure 2:**
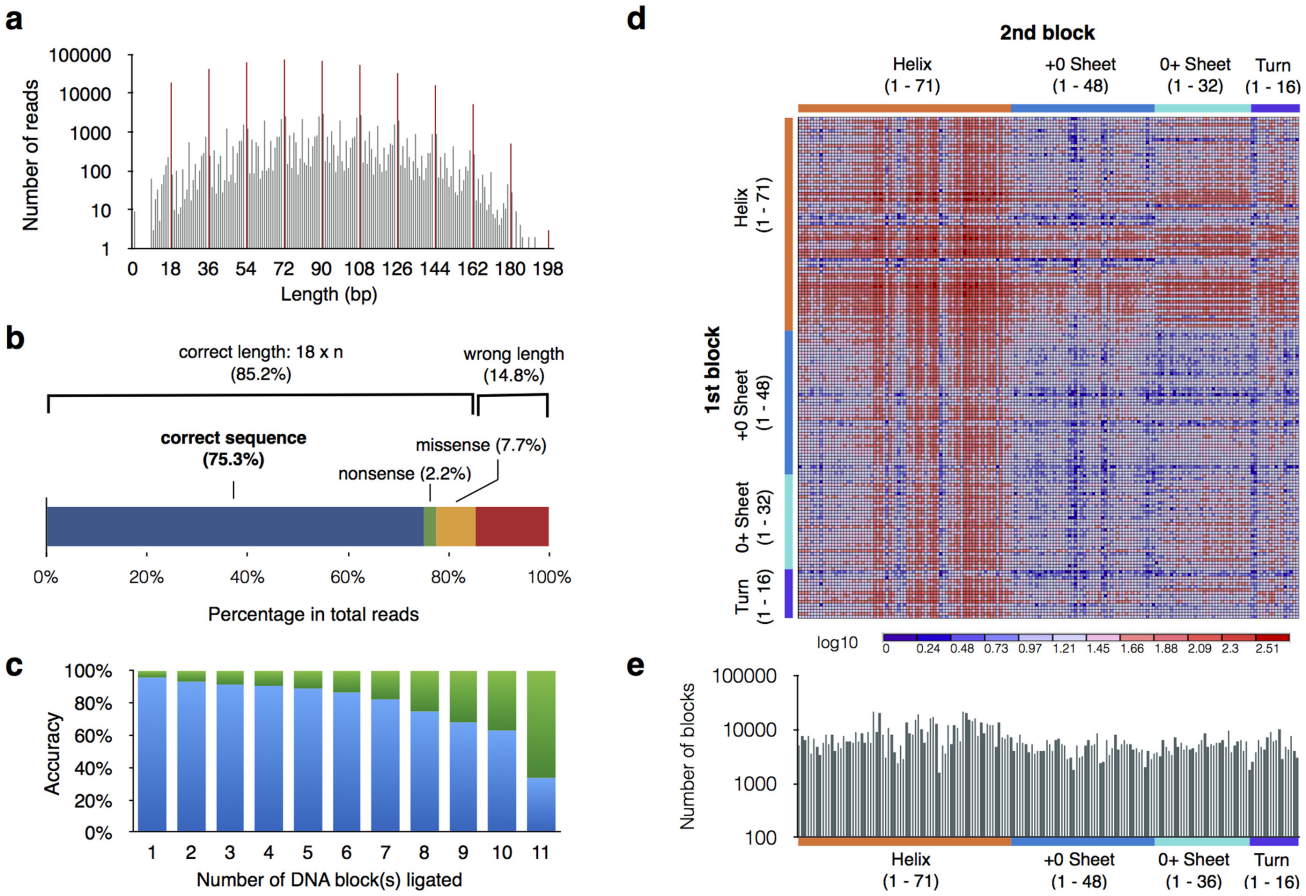
Next-gen sequencing analysis of assembled DNA library. (a) The length distribution of a total of 449,748 high quality DNA sequences extracted from the Miseq reads. Ligated DNA blocks with a correct length of a multiple of 18 bp (red) and others (gray) are represented as a bar graph. (b) Percentage of correct DNA block sequences encoding polypeptides consisting of ten amino acids (75.3%), incorrect sequences with nonsense mutation (2.2%), missense mutation (7.7%) and wrong length (14.8%). (c) Accuracy of assembled gene library per concatemer is calculated based on the portion of accurate (blue) and incorrect (green) sequences. (d) Heat map of the sequence count shows relative abundance of 167 by 167 DNA block combinations represented in the combinatorial gene library (helix 34 was excluded due to the absence in the preparation step). Corresponding protein secondary structure is denoted. (e) Cumulative instances of the representation of each DNA block in the ligated combinatorial gene library.

**Figure 3:**
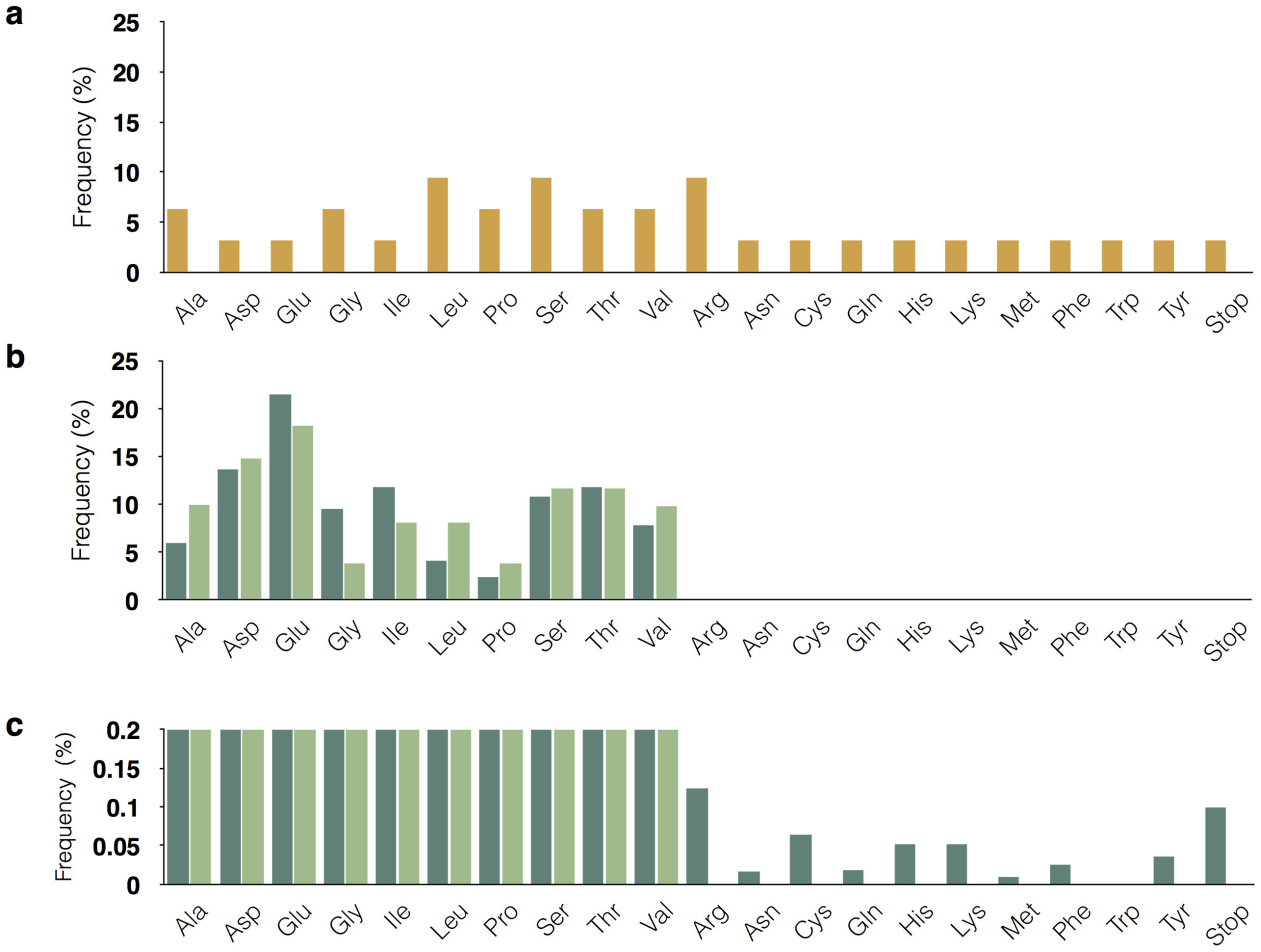
Comparison of amino acid representation between the poly-NNK library and overhang-based random library. (a) Amino acid codon frequency in the traditional poly-NNK DNA library (orange). (b) Amino acid frequency in the initially prepared 8288 DNA blocks limited to ten types of amino acids (light green) and the actual combinatorial gene sequences obtained through overhang-based ligation (dark green). (c) An enlarged view of Fig. 3b with Y axis set from 0 to 0.2%.
