# Supplementary material for: An overhang-based DNA block shuffling method for creating a customized random library
Source: Sci Rep. 2015 May 26;5:9740. doi: 10.1038/srep09740 (PMC4443763; doi:10.1038/srep09740)
Supplement: Supplementary Information — Supplementary materials [file srep09740-s1.pdf]

# An overhang-based DNA block shuffling method for creating a customized random library

Kosuke Fujishima, Chris Venter, Kendrick Wang, Raphael Ferreira and Lynn J. Rothschild

## Supplementary figures and text:

**Supplementary Figure 1 |** Methods for creating a combinatorial gene library

**Supplementary Figure 2 |** Per base sequence quality of Miseq reads

**Supplementary Figure 3 |** Average Quality score distribution of Miseq reads after end-trimming

**Supplementary File 1 |** Perl program for counting and extracting high quality assembled DNA sequences harboring PCR primers

**Supplementary Table 1 |** Estimation of the number of unique genes in the DNA library

**Supplementary Table 2 |** Oligonucleotide sequences for constructing customized random DNA library

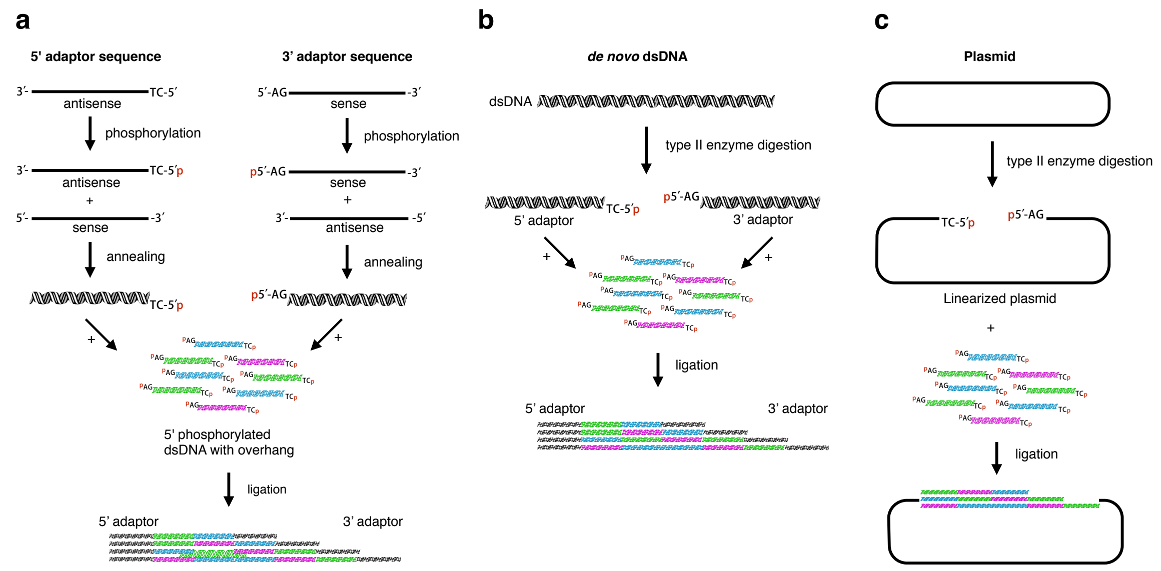


**Supplementary Figure 1 | Methods for creating a combinatorial gene library.** (a) The 5′ and 3′ adaptor sequences are chemically synthesized and annealed to provide desired upstream and downstream sequences. Only the exon side of the dsDNAs is phosphorylated to maintain accurate ligation with exons. (b) Type II enzyme digestion of *de novo* synthesized dsDNA provides long 5′ and 3′ adapter sequences. This is a beneficial approach to perform combinatorial design embedded within a long gene or genetic elements. (c) Type II enzyme digestion of *de novo* and commercially available plasmids serve as a backbone for vector library construction, aiming for a downstream *in vivo* analysis.

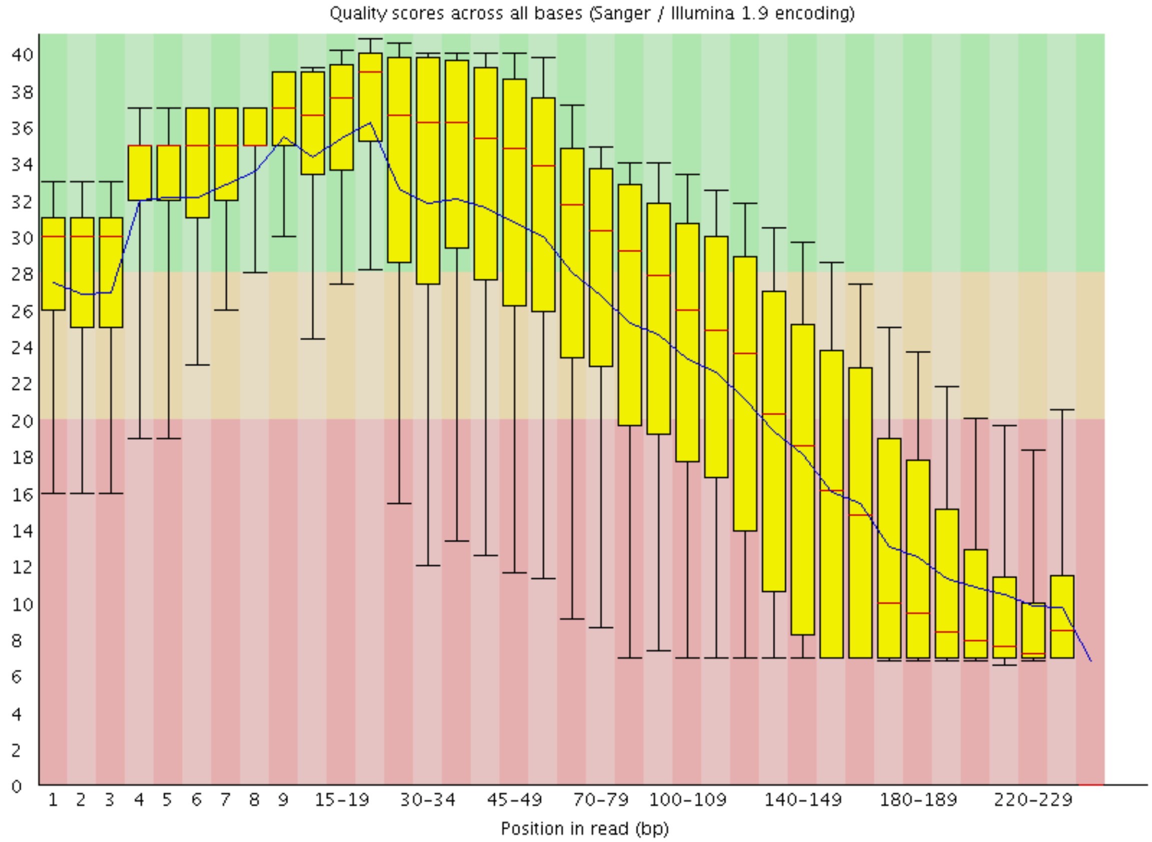


**Supplementary Figure 2 | Per base sequence quality of Miseq reads after end-trimming.** Per base sequence quality was calculated and visualized for total 1,332,848 end-trimmed reads using FastQC package in the Galaxy platform (https://usegalaxy.org). Deterioration toward later cycles, a common feature known as phasing was observed.

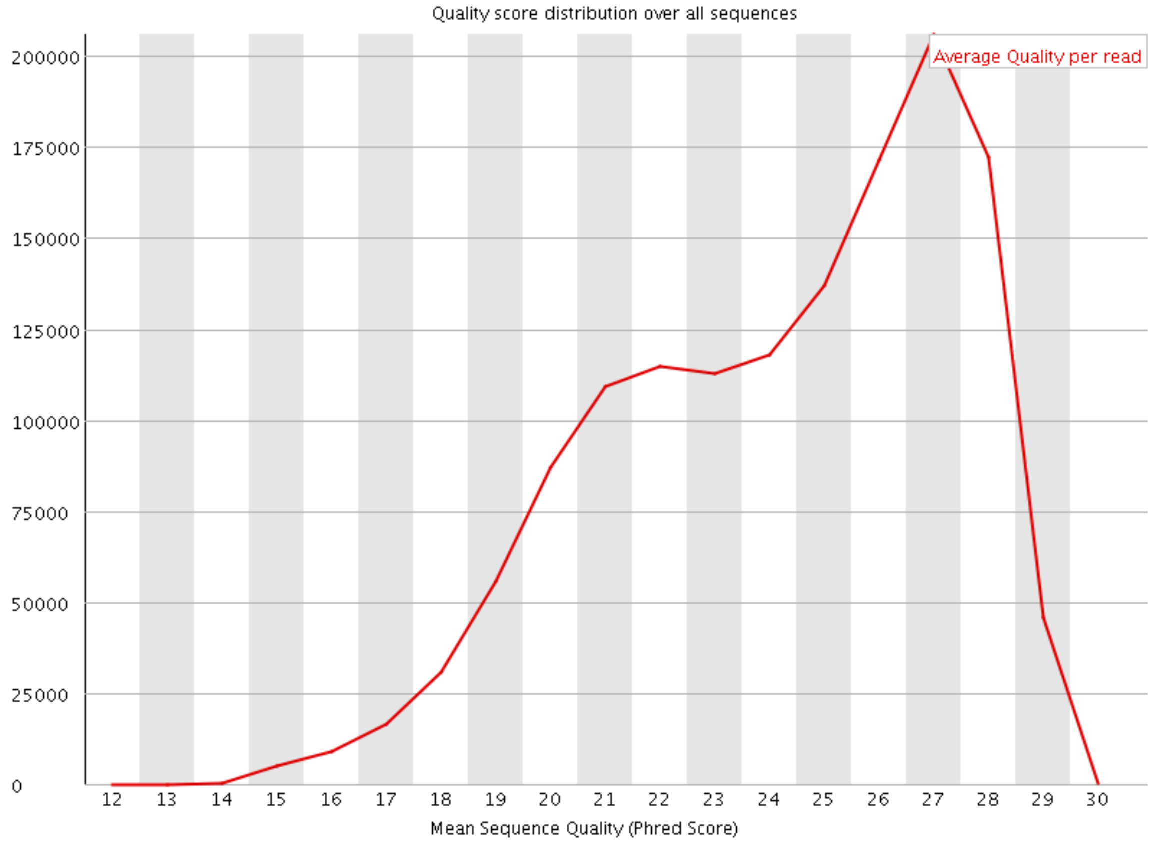


**Supplementary Figure 3 | Average Quality score distribution of Miseq reads after end-trimming.** Mean Phred quality score distribution of total 1,332,848 sequence reads are calculated and visualized using FastQC package in the Galaxy platform after low-quality end trimming. Peak was observed around Q27, while majority of the sequences distributed from Q18 to Q29.

```
#!/usr/bin/perl
use strict;

open(DATA, $ARGV[0]); # input must be Multi-FASTA file
my @ref=();
while(<DATA>){
    if($_=~/^\>/){
       chomp $_;
       my $id = $_;
    }elsif($_=~/GAAGCTTC(\w+)AGTTTAAA/){#sense strand
       my $str = $1;
       my $len=length($str);
       $ref[length($str)]++;
       if($len % 18 == 0){ # ligated DNA block = multiple of 18 bp
       print "$str\n";
       }
       $str="";
    }elsif($_=~/TTTAAACT(\w+)GAAGCTTC/){#reverse strand
       my $r = $1;
       my $lenr=length($r);
       $ref[length($r)]++;
       if($lenr % 18 == 0){ # ligated DNA block = multiple of 18 bp
       print "$r\n";
       $r="";
    }
}

# Counting number of sequences for each length

my $count;
for ($count = 0; $count < 230; $count++){
       print "$count $ref[$count]\n";
}
```

**Supplementary File 1 | Perl program for counting and extracting high quality assembled DNA sequences containing adapters.** Multi-FASTA file containing 1,332,848 reads were filtered using this Perl script to extract total of 449,748 ligated DNA blocks with high sequence quality that harbor 5′ and 3′ adapter sequences (at least 8 bp complete match on each ends). It also returns the number of reads per length as shown in Figure 2a.

| Number of ligated DNA block(s) | 2 | 3 | 4 | 5 | 6 | 7 | 8 | 9 | 10 | 11 |
|---|---|---|---|---|---|---|---|---|---|---|
| A) Sequence reads | 40425 | 59129 | 66429 | 62967 | 46436 | 28105 | 12289 | 3610 | 325 | 1 |
| B) Non-redundant sequence reads | 39890 (98.7%) | 58905 (99.6%) | 66249 (99.7%) | 62799 (99.9%) | 46386 (99.9%) | 28086 (99.9%) | 12287 (99.9%) | 3610 (100%) | 325 (100%) | 1 (100%) |
| C) Total number of DNA molecules | 5.6E+12 | 1.2E+13 | 1.8E+13 | 2.2E+13 | 1.9E+13 | 1.4E+13 | 6.8E+12 | 2.2E+12 | 2.2E+11 | 7.6E+08 |
| D) DNA block combinations (Theoretical) | 6.9E+07 | 5.7E+11 | 4.7E+15 | 3.9E+19 | 3.2E+23 | 2.7E+27 | 2.2E+31 | 1.8E+35 | 1.5E+39 | 1.3E+43 |
| E) Unique genes | **6.8E+07** | **5.7E+11** | **1.8E+13** | **2.2E+13** | **1.9E+13** | **1.4E+13** | **6.8E+12** | **2.2E+12** | **2.2E+11** | **7.6E+08** |

**Supplementary Table 1 | Estimation of the number of unique genes in the DNA library.** For each ligated concatemer (2 to 11 blocks in length), we calculated the number of A) sequence reads with correct length, B) non-redundant sequences with relative portion to A shown in bracket, C) number of DNA molecules, assuming that assembled DNA library consist of total $10^{14}$ DNA blocks, D) theoretical DNA block combination that follows $N = 8288^n$, where n is the number of ligated blocks, and E) estimated unique genes, where short concatemer less than four (left side of blue line) reach the upper limit of DNA block combination as shown in D, while longer concatemers are almost equivalent to the total number of DNA molecules represented in C, due to their low redundancy.

**Supplementary Table 2  Oligonucleotide sequences for constructing customized random DNA library**

| Name | Forward sequence | Reverse sequence |
|---|---|---|
| 5' Adaptor | GGAGCGATCGCCATGGAAGCTTC | CTGAAGCTTCCATGGCGATCGCTCC |
| 3' Adaptor | AGTTTAAACTAGCATATGCGG | CCGCATATGCTAGTTTAAA |
| Primers for PCR amplification | GGAGCGATCGCCATGGAA | CCGCATATGCTAGTTTAAACT |
| Helix-1 | AGASSYCGASGASSYCGA | CTTCGRSSTCSTCGRSST |
| Helix-2 | AGASSYCGASGASSYCWC | CTGWGRSSTCSTCGRSST |
| Helix-3 | AGASSYCGASGASATCGA | CTTCGATSTCSTCGRSST |
| Helix-4 | AGASSYCGASGASATCWC | CTGWGATSTCSTCGRSST |
| Helix-5 | AGASSYCGASGASGGCGA | CTTCGCCSTCSTCGRSST |
| Helix-6 | AGASSYCGASGASGGCWC | CTGWGCCSTCSTCGRSST |
| Helix-7 | AGASSYCGASWCASYCGA | CTTCGRSTGWSTCGRSST |
| Helix-8 | AGASSYCGASWCASYCWC | CTGWGRSTGWSTCGRSST |
| Helix-9 | AGASSYCGASWCAATCGA | CTTCGATTGWSTCGRSST |
| Helix-10 | AGASSYCGASWCAATCWC | CTGWGATTGWSTCGRSST |
| Helix-11 | AGASSYCGASWCAGGCGA | CTTCGCCTGWSTCGRSST |
| Helix-12 | AGASSYCGASWCAGGCWC | CTGWGCCTGWSTCGRSST |
| Helix-13 | AGASSYCWCAGASSYCGA | CTTCGRSSTCTGWGRSST |
| Helix-14 | AGASSYCWCAGASSYCWC | CTGWGRSSTCTGWGRSST |
| Helix-15 | AGASSYCWCAGASATCGA | CTTCGATSTCTGWGRSST |
| Helix-16 | AGASSYCWCAGASATCWC | CTGWGATSTCTGWGRSST |
| Helix-17 | AGASSYCWCAGASGGCGA | CTTCGCCSTCTGWGRSST |
| Helix-18 | AGASSYCWCAGASGGCWC | CTGWGCCSTCTGWGRSST |
| Helix-19 | AGASSYCWCAWCASYCGA | CTTCGRSTGWTGWGRSST |
| Helix-20 | AGASSYCWCAWCASYCWC | CTGWGRSTGWTGWGRSST |
| Helix-21 | AGASSYCWCAWCAATCGA | CTTCGATTGWTGWGRSST |
| Helix-22 | AGASSYCWCAWCAATCWC | CTGWGATTGWTGWGRSST |
| Helix-23 | AGASSYCWCAWCAGGCGA | CTTCGCCTGWTGWGRSST |
| Helix-24 | AGASSYCWCAWCAGGCWC | CTGWGCCTGWTGWGRSST |
| Helix-25 | AGASGGCGASGASSYCGA | CTTCGRSSTCSTCGCCST |
| Helix-26 | AGASGGCGASGASSYCWC | CTGWGRSSTCSTCGCCST |
| Helix-27 | AGASGGCGASGASATCGA | CTTCGATSTCSTCGCCST |
| Helix-28 | AGASGGCGASGASATCWC | CTGWGATSTCSTCGCCST |
| Helix-29 | AGASGGCGASGASGGCGA | CTTCGCCSTCSTCGCCST |
| Helix-30 | AGASGGCGASGASGGCWC | CTGWGCCSTCSTCGCCST |
| Helix-31 | AGASGGCGASWCASYCGA | CTTCGRSTGWSTCGCCST |
| Helix-32 | AGASGGCGASWCASYCWC | CTGWGRSTGWSTCGCCST |
| Helix-33 | AGASGGCGASWCAATCGA | CTTCGATTGWSTCGCCST |
| Helix-34 | AGASGGCGASWCAATCWC | CTGWGATTGWSTCGCCST |
| Helix-35 | AGASGGCGASWCAGGCGA | CTTCGCCTGWSTCGCCST |
| Helix-36 | AGASGGCGASWCAGGCWC | CTGWGCCTGWSTCGCCST |
| Helix-37 | AGASGGCWCAGASSYCGA | CTTCGRSSTCTGWGCCST |

| | | |
|---|---|---|
| Helix-38 | AGASGGCWCAGASSYCWC | CTGWGRSSTCTGWGCCST |
| Helix-39 | AGASGGCWCAGASATCGA | CTTCGATSTCTGWGCCST |
| Helix-40 | AGASGGCWCAGASATCWC | CTGWGATSTCTGWGCCST |
| Helix-41 | AGASGGCWCAGASGGCGA | CTTCGCCSTCTGWGCCST |
| Helix-42 | AGASGGCWCAGASGGCWC | CTGWGCCSTCTGWGCCST |
| Helix-43 | AGASGGCWCAWCASYCGA | CTTCGRSTGWTGWGCCST |
| Helix-44 | AGASGGCWCAWCASYCWC | CTGWGRSTGWTGWGCCST |
| Helix-45 | AGASGGCWCAWCAATCGA | CTTCGATTGWTGWGCCST |
| Helix-46 | AGASGGCWCAWCAATCWC | CTGWGATTGWTGWGCCST |
| Helix-47 | AGASGGCWCAWCAGGCGA | CTTCGCCTGWTGWGCCST |
| Helix-48 | AGASGGCWCAWCAGGCWC | CTGWGCCTGWTGWGCCST |
| Helix-49 | AGASATCGASGASSYCGA | CTTCGRSSTCSTCGATST |
| Helix-50 | AGASATCGASGASSYCWC | CTGWGRSSTCSTCGATST |
| Helix-51 | AGASATCGASGASATCGA | CTTCGATSTCSTCGATST |
| Helix-52 | AGASATCGASGASATCWC | CTGWGATSTCSTCGATST |
| Helix-53 | AGASATCGASGASGGCGA | CTTCGCCSTCSTCGATST |
| Helix-54 | AGASATCGASGASGGCWC | CTGWGCCSTCSTCGATST |
| Helix-55 | AGASATCGASWCASYCGA | CTTCGRSTGWSTCATST |
| Helix-56 | AGASATCGASWCASYCWC | CTGWGRSTGWSTCGATST |
| Helix-57 | AGASATCGASWCAATCGA | CTTCGATTGWSTCGATST |
| Helix-58 | AGASATCGASWCAATCWC | CTGWGATTGWSTCGATST |
| Helix-59 | AGASATCGASWCAGGCGA | CTTCGCCTGWSTCGATST |
| Helix-60 | AGASATCGASWCAGGCWC | CTGWGCCTGWSTCGATST |
| Helix-61 | AGASATCWCAGASSYCGA | CTTCGRSSTCTGWGATST |
| Helix-62 | AGASATCWCAGASSYCWC | CTGWGRSSTCTGWGATST |
| Helix-63 | AGASATCWCAGASATCGA | CTTCGATSTCTGWGATST |
| Helix-64 | AGASATCWCAGASATCWC | CTGWGATSTCTGWGATST |
| Helix-65 | AGASATCWCAGASGGCGA | CTTCGCCSTCTGWGATST |
| Helix-66 | AGASATCWCAGASGGCWC | CTGWGCCSTCTGWGATST |
| Helix-67 | AGASATCWCAWCASYCGA | CTTCGRSTGWTGWGATST |
| Helix-68 | AGASATCWCAWCASYCWC | CTGWGRSTGWTGWGATST |
| Helix-69 | AGASATCWCAWCAATCGA | CTTCGATTGWTGWGATST |
| Helix-70 | AGASATCWCAWCAATCWC | CTGWGATTGWTGWGATST |
| Helix-71 | AGASATCWCAWCAGGCGA | CTTCGCCTGWTGWGATST |
| Helix-72 | AGASATCWCAWCAGGCWC | CTGWGCCTGWTGWGATST |
| | | |
| +0Sheet-1 | AGASGYCGASGYCGASSY | CTRSSTCGRCSTCGRCST |
| +0Sheet-2 | AGASGYCGASGYCWCASY | CTRSTGWGRCSTCGRCST |
| +0Sheet-3 | AGASGYCGASMTCGASSY | CTRSSTCGAKSTCGRCST |
| +0Sheet-4 | AGASGYCGASMTCWCASY | CTRSTGWGAKSTCGRCST |
| +0Sheet-5 | AGASGYCWCAGYCGASSY | CTRSSTCGRCTGWGRCST |
| +0Sheet-6 | AGASGYCWCAGYCWCASY | CTRSTGWGRCTGWGRCST |
| +0Sheet-7 | AGASGYCWCAMTCGASSY | CTRSSTCGAKTGWGRCST |

| | | |
|---|---|---|
| +0Sheet-8 | AGASGYCWCAMTCWCASY | CTRSTGWGAKTGWGRCST |
| +0Sheet-9 | AGASMTCGASGYCGASSY | CTRSSTCGRCSTCGAKST |
| +0Sheet-10 | AGASMTCGASGYCWCASY | CTRSTGWGRCSTCGAKST |
| +0Sheet-11 | AGASMTCGASMTCGASSY | CTRSSTCGAKSTCGAKST |
| +0Sheet-12 | AGASMTCGASMTCWCASY | CTRSTGWGAKSTCGAKST |
| +0Sheet-13 | AGASMTCWCAGYCGASSY | CTRSSTCGRCTGWGAKST |
| +0Sheet-14 | AGASMTCWCAGYCWCASY | CTRSTGWGRCTGWGAKST |
| +0Sheet-15 | AGASMTCWCAMTCGASSY | CTRSSTCGAKTGWGAKST |
| +0Sheet-16 | AGASMTCWCAMTCWCASY | CTRSTGWGAKTGWGAKST |
| +0Sheet-17 | AGASGYCGASGYCGASAT | CTTASTCGRCSTCGRCST |
| +0Sheet-18 | AGASGYCGASGYCWCAAT | CTTATGWGRCSTCGRCST |
| +0Sheet-19 | AGASGYCGASMTCGASAT | CTTASTCGAKSTCGRCST |
| +0Sheet-20 | AGASGYCGASMTCWCAAT | CTTATGWGAKSTCGRCST |
| +0Sheet-21 | AGASGYCWCAGYCGASAT | CTTASTCGRCTGWGRCST |
| +0Sheet-22 | AGASGYCWCAGYCWCAAT | CTTATGWGRCTGWGRCST |
| +0Sheet-23 | AGASGYCWCAMTCGASAT | CTTASTCGAKTGWGRCST |
| +0Sheet-24 | AGASGYCWCAMTCWCAAT | CTTATGWGAKTGWGRCST |
| +0Sheet-25 | AGASMTCGASGYCGASAT | CTTASTCGRCSTCGAKST |
| +0Sheet-26 | AGASMTCGASGYCWCAAT | CTTATGWGRCSTCGAKST |
| +0Sheet-27 | AGASMTCGASMTCGASAT | CTTASTCGAKSTCGAKST |
| +0Sheet-28 | AGASMTCGASMTCWCAAT | CTTATGWGAKSTCGAKST |
| +0Sheet-29 | AGASMTCWCAGYCGASAT | CTTASTCGRCTGWGAKST |
| +0Sheet-30 | AGASMTCWCAGYCWCAAT | CTTATGWGRCTGWGAKST |
| +0Sheet-31 | AGASMTCWCAMTCGASAT | CTTASTCGAKTGWGAKST |
| +0Sheet-32 | AGASMTCWCAMTCWCAAT | CTTATGWGAKTGWGAKST |
| +0Sheet-33 | AGASGYCGASGYCGASGG | CTCCSTCGRCSTCGRCST |
| +0Sheet-34 | AGASGYCGASGYCWCAGG | CTCCTGWGRCSTCGRCST |
| +0Sheet-35 | AGASGYCGASMTCGASGG | CTCCSTCGAKSTCGRCST |
| +0Sheet-36 | AGASGYCGASMTCWCAGG | CTCCTGWGAKSTCGRCST |
| +0Sheet-37 | AGASGYCWCAGYCGASGG | CTCCSTCGRCTGWGRCST |
| +0Sheet-38 | AGASGYCWCAGYCWCAGG | CTCCTGWGRCTGWGRCST |
| +0Sheet-39 | AGASGYCWCAMTCGASGG | CTCCSTCGAKTGWGRCST |
| +0Sheet-40 | AGASGYCWCAMTCWCAGG | CTCCTGWGAKTGWGRCST |
| +0Sheet-41 | AGASMTCGASGYCGASGG | CTCCSTCGRCSTCGAKST |
| +0Sheet-42 | AGASMTCGASGYCWCAGG | CTCCTGWGRCSTCGAKST |
| +0Sheet-43 | AGASMTCGASMTCGASGG | CTCCSTCGAKSTCGAKST |
| +0Sheet-44 | CTCCSTCGAKSTCGAKST | CTCCTGWGAKSTCGAKST |
| +0Sheet-45 | AGASMTCWCAGYCGASGG | CTCCSTCGRCTGWGAKST |
| +0Sheet-46 | AGASMTCWCAGYCWCAGG | CTCCTGWGRCTGWGAKST |
| +0Sheet-47 | AGASMTCWCAMTCGASGG | CTCCSTCGAKTGWGAKST |
| +0Sheet-48 | AGASMTCWCAMTCWCAGG | CTCCTGWGAKTGWGAKST |
| | | |
| 0+Sheet-1 | AGYCGASGYCGASGYCGA | CTTCGRCSTCGRCSTCGR |

| | | |
|---|---|---|
| 0+Sheet-2 | AGYCGASGYCGASGYCWC | CTGWGRCSTCGRCSTCGR |
| 0+Sheet-3 | AGYCGASGYCGASMTCGA | CTTCGAKSTCGRCSTCGR |
| 0+Sheet-4 | AGYCGASGYCGASMTCWC | CTGWGAKSTCGRCSTCGR |
| 0+Sheet-5 | AGYCGASGYCWCAGYCGA | CTTCGRCTGWGRCSTCGR |
| 0+Sheet-6 | AGYCGASGYCWCAGYCWC | CTGWGRCTGWGRCSTCGR |
| 0+Sheet-7 | AGYCGASGYCWCAMTCGA | CTTCGAKTGWGRCSTCGR |
| 0+Sheet-8 | AGYCGASGYCWCAMTCWC | CTGWGAKTGWGRCSTCGR |
| 0+Sheet-9 | AGYCGASMTCGASGYCGA | CTTCGRCSTCGAKSTCGR |
| 0+Sheet-10 | AGYCGASMTCGASGYCWC | CTGWGRCSTCGAKSTCGR |
| 0+Sheet-11 | AGYCGASMTCGASMTCGA | CTTCGAKSTCGAKSTCGR |
| 0+Sheet-12 | AGYCGASMTCGASMTCWC | CTGWGAKSTCGAKSTCGR |
| 0+Sheet-13 | AGYCGASMTCWCAGYCGA | CTTCGRCTGWGAKSTCGR |
| 0+Sheet-14 | AGYCGASMTCWCAGYCWC | CTGWGRCTGWGAKSTCGR |
| 0+Sheet-15 | AGYCGASMTCWCAMTCGA | CTTCGAKTGWGAKSTCGR |
| 0+Sheet-16 | AGYCGASMTCWCAMTCWC | CTGWGAKTGWGAKSTCGR |
| 0+Sheet-17 | AGYCWCAGYCGASGYCGA | CTTCGRCSTCGRCTGWGR |
| 0+Sheet-18 | AGYCWCAGYCGASGYCWC | CTGWGRCSTCGRCTGWGR |
| 0+Sheet-19 | AGYCWCAGYCGASMTCGA | CTTCGAKSTCGRCTGWGR |
| 0+Sheet-20 | AGYCWCAGYCGASMTCWC | CTGWGAKSTCGRCTGWGR |
| 0+Sheet-21 | AGYCWCAGYCWCAGYCGA | CTTCGRCTGWGRCTGWGR |
| 0+Sheet-22 | AGYCWCAGYCWCAGYCWC | CTGWGRCTGWGRCTGWGR |
| 0+Sheet-23 | AGYCWCAGYCWCAMTCGA | CTTCGAKTGWGRCTGWGR |
| 0+Sheet-24 | AGYCWCAGYCWCAMTCWC | CTGWGAKTGWGRCTGWGR |
| 0+Sheet-25 | AGYCWCAMTCGASGYCGA | CTTCGRCSTCGAKTGWGR |
| 0+Sheet-26 | AGYCWCAMTCGASGYCWC | CTGWGRCSTCGAKTGWGR |
| 0+Sheet-27 | AGYCWCAMTCGASMTCGA | CTTCGAKSTCGAKTGWGR |
| 0+Sheet-28 | AGYCWCAMTCGASMTCWC | CTGWGAKSTCGAKTGWGR |
| 0+Sheet-29 | AGYCWCAMTCWCAGYCGA | CTTCGRCTGWGAKTGWGR |
| 0+Sheet-30 | AGYCWCAMTCWCAGYCWC | CTGWGRCTGWGAKTGWGR |
| 0+Sheet-31 | AGYCWCAMTCWCAMTCGA | CTTCGAKTGWGAKTGWGR |
| 0+Sheet-32 | AGYCWCAMTCWCAMTCWC | CTGWGAKTGWGAKTGWGR |
| turn-1 | AGASGSTCCTGGCGYCGA | CTTCGRCGCCAGGASCST |
| turn-2 | AGASGSTCCTGGCGYCWC | CTGWGRCGCCAGGASCST |
| turn-3 | AGASGSTCCTGGCMTCGA | CTTCGAKGCCAGGASCST |
| turn-4 | AGASGSTCCTGGCMTCWC | CTGWGAKGCCAGGASCST |
| turn-5 | AGASGSTGGCCCTGYCGA | CTTCGRCAGGGCCASCST |
| turn-6 | AGASGSTGGCCCTGYCWC | CTGWGRCAGGGCCASCST |
| turn-7 | AGASGSTGGCCCTMTCGA | CTTCGAKAGGGCCASCST |
| turn-8 | AGASGSTGGCCCTMTCWC | CTGWGAKAGGGCCASCST |
| turn-9 | AGYCKCACCTGGCGASGY | CTRCSTCGCCAGGTGMGR |
| turn-10 | AGYCKCACCTGGCGASMT | CTAKSTCGCCAGGTGMGR |
| turn-11 | AGYCKCACCTGGCWCAGY | CTRCTGWGCCAGGTGMGR |

| | | |
|---|---|---|
| turn-12 | AGYCKCACCTGGCWCAMT | CTAKTGWGCCAGGTGMGR |
| turn-13 | AGYCKCAGGCCCTGASGY | CTRCSTCAGGGCCTGMGR |
| turn-14 | AGYCKCAGGCCCTGASMT | CTAKSTCAGGGCCTGMGR |
| turn-15 | AGYCKCAGGCCCTWCAGY | CTRCTGWAGGGCCTGMGR |
| turn-16 | AGYCKCAGGCCCTWCAMT | CTAKTGWAGGGCCTGMGR |
